# Impact of posterior septectomy on olfaction in endoscopic endonasal transsphenoidal surgery

**DOI:** 10.1371/journal.pone.0316263

**Published:** 2025-01-02

**Authors:** Jae Yoon Lee, Jae Sung Park, Sin Soo Jeun, Sung Won Kim, Do Hyun Kim, Soo Whan Kim

**Affiliations:** 1 Department of Otolaryngology-Head and Neck Surgery, Seoul Saint Mary’s Hospital, College of Medicine, The Catholic University of Korea, Seoul, Korea; 2 Department of Neurosurgery, Seoul Saint Mary’s Hospital, College of Medicine, The Catholic University of Korea, Seoul, Korea; University Putra Malaysia, MALAYSIA

## Abstract

**Background:**

Endoscopic endonasal transsphenoidal surgery is widely used to resect pituitary adenomas, yet its impact on olfactory function after resection of the posterosuperior nasal septum remains a concern. To optimize surgical techniques to preserve olfactory function, it is essential to understand the relationship between the extent of septal resection and olfactory outcomes.

**Methods:**

This retrospective study analyzed 295 patients who underwent pituitary adenoma surgery. The extent of nasal septum resection was quantified and its impact on olfactory function was assessed using the Cross-Cultural Smell Identification Test (CCSIT), Sino-Nasal Outcome Test-22 (SNOT-22), and a Visual Analog Scale (VAS) for olfactory loss. Preoperative and 6-month postoperative scores were compared to evaluate changes in olfactory function.

**Results:**

There was a significant correlation between larger septal resections and greater reductions in CCSIT scores, indicating a decline in olfactory function. Furthermore, patients with more extensive septal resections reported increased discomfort and olfactory loss, as evidenced by higher SNOT-22 and VAS scores. These findings highlight the importance of the nasal septum in maintaining laminar airflow and its role in olfactory function.

**Conclusion:**

Study underscores the adverse effects of extensive posterior septectomy on olfactory outcomes. Minimizing the extent of septal resection may help preserve olfactory function, suggesting a need for surgical strategies that maintain septum integrity to reduce the risk of postoperative olfactory impairment.

## Introduction

The advent of endoscopic endonasal transsphenoidal surgery revolutionized the treatment of sellar and parasellar tumors, offering a minimally invasive approach with enhanced visualization and reduced morbidity compared to traditional techniques [1]. Despite its numerous advantages, this surgery harbors inherent risks, particularly concerning olfactory function, owing to the obligatory resection of nasal structures [2]. The olfactory neuroepithelium, located in the superior portion of the nasal vault, is integral to the sense of smell [3], and its proximity to the surgical field makes it susceptible to iatrogenic injury during such procedures.

Preservation of the olfactory strip is crucial to maintain olfactory function during surgical procedures [4]. Studies have documented the impact of endoscopic endonasal surgery on olfactory outcomes [5], prompting a shift towards the adoption of minimally invasive strategies aimed at preserving olfaction [6–8]. Despite efforts by several groups to minimize the impact on olfactory function through various techniques, the precise effects of structural changes within the nasal cavity on postoperative olfaction remain largely unexplored. While a cadaveric study suggested that posterior septectomy influences the sinonasal quality of life, this needs clinical validation [9].

Therefore, we quantified the extent of posterior septectomy in patients and correlated it with changes in olfactory function between before and after surgery.

## Materials and methods

This retrospective investigation assessed the outcomes of patients who underwent endoscopic endonasal transsphenoidal surgery from March 2010 through December 2022. The study was approved by the Institutional Review Board of Seoul Saint Mary’s Hospital (KC20OISI0461), which waived the need for informed consent from patients given the retrospective study design.

Sinonasal functionality was evaluated utilizing a suite of tests: the Connecticut Chemosensory Clinical Research Center (CCCRC) test [10], Cross-Cultural Smell Identification Test (CCSIT) [11], Sino-Nasal Outcome Test-22 (SNOT-22) [12], and a Visual Analog Scale (VAS), before surgery and 6 months following the procedure. Changes in sinonasal outcomes were determined by deducting the preoperative scores from the postoperative ones, with CCCRC outcomes averaged across both sides.

The research team consisted of two independent neurosurgeons and two rhinology experts, focusing exclusively on pituitary adenomas to minimize the variability associated with specific surgical techniques. All interventions used a bilateral transnasal approach, incorporating a modified nasoseptal rescue flap, and involved partial resection of the posterior nasal septum, including the perpendicular plate of the ethmoid bone, the vomer, and the sphenoid sinus anterior wall [13].

**Fig 1:**
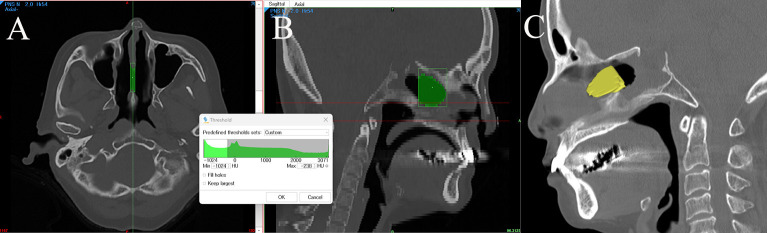
Quantification of the resected posterior septal area using computed tomography. A. Reconstructed image of the resected area (yellow) in a sagittal view of the nasal septum. B. Measuring the extent of posterior septectomy using Mimics software.

The resected septal area was quantified using three-dimensional reconstruction of CT scans of the bony nasal septum, using Mimics Base software (ver. 22, Materialise, Leuven, Belgium). Following the methodology similar to Kim et al., the measurement process began with identifying the axial image showing the most prominent septal defect area (Fig 1A) [14]. The boundary delineation utilized the differential brightness between subcutaneous tissue and cartilage, which typically shows a mean difference of 50 hounsfield units. In the axial view, the margin along the defect was marked with dots to create an initial boundary. When viewing this marked area in the sagittal reconstructed image (Fig 1B), the area initially included the sphenoid sinus cavity. To accurately measure only the surgically resected septum area, the sphenoid sinus cavity was excluded by referring to the patient’s preoperative CT. The posterior boundary for measurements was defined by the preoperative anterior wall of the sphenoid sinus, as inferred from the bone contour remaining after the surgery. This step was critical as the standard surgical technique typically involves resecting approximately 1–2 cm of the posterior septum measured from the anterior sphenoid wall [15]. The anterior margin of the septectomy area was further detailed along the midline of the septum air-soft tissue interface. The final three-dimensionally reconstructed area (Fig 1C) was verified against the sagittal image to ensure accurate representation of the septectomy site. The total septectomy area was calculated by averaging measurements from both sagittal surfaces of the reconstructed area, providing a more reliable estimate of the actual resection size.

Statistical comparisons were made using the Student’s *t*-test, with all analyses performed using IBM SPSS Statistics (ver. 24.0, SPSS, Chicago, IL, USA). A p-value of less than 0.05 was considered statistically significant.

## Results

Table 1 summarizes the baseline preoperative characteristics of the 295 patients. A nasoseptal flap was used to repair cerebrospinal fluid leaks in 7.8%, which is within the reported range [16, 17]. Baseline values of the olfactory function tests and questionnaire scores were also within the normal ranges [10–12, 18].

**Table 1:** Baseline characteristics.

	All patients (*n* = 295)
Sex	
Male	148 (50.2%)
Female	147 (49.8%)
Age (years)	51.29 ± 15.48
Fat graft	12 (4.1%)
Nasoseptal flap	23 (7.8%)
CCCRC	8.12 ± 2.35
CCSIT	8.70 ± 2.26
NOSE	3.31 ± 4.30
SNOT-22	15.64 ± 16.44
VAS	
Nasal stuffiness	1.87 ± 2.16
Sneezing	2.32 ± 2.36
Rhinorrhea	2.21 ± 2.43
Snoring	3.33 ± 2.96
Headache	3.24 ± 3.02
Facial pain	1.35 ± 2.00
Loss of smell	1.47 ± 2.08

The table shows the mean SD for continuous variables, number (%) for binary variables, and number per group for categorical variables. The butanol threshold test for both sides are averaged. CCCRC, Connecticut Chemosensory Clinical Research Center test; CCSIT, cross cultural smell identification test; NOSE, nasal obstruction symptom evaluation; SNOT, sinonasal outcome test; VAS, visual analogue scale

Table 2 shows there was a significant correlation between the extent of the resected posterior septal area and reduction in CCSIT scores. The results of the CCCRC showed a similar trend, but did not reach statistical significance.

**Table 2:** Factors associated with posterior septectomy area.

	Correlation coefficient	*p* value
Δ CCCRC	–0.095	0.102
Δ CCSIT	–0.142	0.015*
Δ NOSE	0.113	0.053
Δ SNOT-22	0.172	0.003**
Δ VAS		
Nasal stuffiness	0.071	0.225
Sneezing	0.104	0.076
Rhinorrhea	0.099	0.089
Snoring	0.029	0.617
Headache	0.093	0.112
Facial pain	0.03	0.612
Loss of smell	0.121	0.037*

Changes in sinonasal outcomes are shown as delta (Δ), indicating the postoperative value minus the preoperative value. The CCCRC results for both sides were averaged. CCCRC, Connecticut Chemosensory Clinical Research Center test; CCSIT, Cross-Cultural Smell Identification Test; NOSE, Nasal Obstruction Symptom Evaluation; SNOT, Sinonasal Outcome Test; VAS, visual analogue scale

*p<0.05; **p<0.001

This means that for subjective symptoms, patients who undergo more extensive posterior septectomies reported significantly greater discomfort, quantified by elevated SNOT-22 scores. Notably, a larger decrease in olfactory function, assessed with the VAS, was significantly associated with an increased area of posterior septum resected.

## Discussion

Olfactory function is an essential postsurgical sinonasal outcome and its significance is often underestimated [19]. Postoperative olfactory dysfunction significantly reduces a patient’s quality of life, manifesting as an inability to detect spoiled foods, gas leaks, or smoke, along with reduced enjoyment of culinary activities [20]. The olfactory neuroepithelium, which houses sensory receptors crucial for olfaction, is located in the upper nasal vault along the cribriform plate, superior turbinate, and superior septum [21]. In the transnasal transsphenoidal approach, the posterior segment of the septum is resected to ensure sufficient access to the sellar region [22]. Despite concerted efforts to conserve this critical area [6–8], the integrity of the neuroepithelium is often compromised while reaching the sellar floor via transsphenoidal approaches, leading to potential olfactory impairment.

Previous studies have documented marked declines in both the CCCRC and CCSIT scores following endoscopic endonasal transsphenoidal procedures [23]. Similarly, Tam *et al*. observed a significant reduction in the University of Pennsylvania Smell Identification Test scores after surgery, irrespective of the use of a septal flap [24]. Despite these insights, no quantitative analysis has explored the direct relationship between specific anatomical changes and the extent of postoperative olfactory dysfunction.

Therefore, we examined whether extensive manipulation of the posterosuperior nasal septum exacerbates olfactory dysfunction. Using advanced image-reconstruction techniques, we precisely quantified the resected septal area. This revealed a correlation between the magnitude of septal resection and both the severity of olfactory complaints and the deterioration of objective olfactory assessments. The dimensions of the posterosuperior septal defect are crucial for regulating airflow within the nasal cavity [25]. The surgical removal of anatomical structures induces turbulent airflow, leading to nasal dryness and crust formation [26]. Airflow turbulence in the uppermost nasal cavity impedes the stable retention of odorant molecules within the olfactory cleft, thereby curtailing their interaction with the sensory epithelium [3]. Therefore, we hypothesized that the extent of posterior septectomy would directly affect postoperative olfactory outcomes. Our subsequent analysis validated this hypothesis, demonstrating that a larger resected area of the posterior septum is associated with a more significant reduction in olfactory function after surgery.

The observed correlation between extensive posterior septectomy and olfactory dysfunction may be attributable to the significant change in airflow dynamics around the olfactory cleft, a consequence of the integral role of the nasal septum in maintaining laminar airflow and by extension, the equilibrium of the nasal cavity [27]. Such disturbances are exacerbated by an increase in airflow velocity and resultant dryness of the olfactory mucosa, which collectively impede the efficient interaction between sensory receptors and odorants, crucial for olfactory perception [28]. Further research should elucidate the precise mechanisms underlying this association.

In this study, we confirmed that a single factor, the degree of posterior septectomy, plays an important role in postoperative olfactory function. Nevertheless, this study had several limitations. First, the size and characteristics of the tumor inherently influence both the duration and intricacies of the surgical procedure, which in turn may affect postoperative olfactory outcomes. To overcome this problem, we tried to standardize the surgical technique and control variables by limiting the results to patients diagnosed with pituitary adenoma who underwent surgery. Second, the involvement of two neurosurgeons introduces the potential for variability in sinonasal outcomes due to differences in individual surgical expertise and approach. Another consideration is the influence of preoperative olfactory function, which can vary with patient age, potentially confounding the assessment of olfactory outcomes. The age factor is particularly relevant given that a case-control study of 60 pediatric patients showed no significant long-term differences in sniffin’ sticks test results between patients with and without a history of endoscopic endonasal skull base surgery [29].

In the context of endoscopic endonasal skull base surgery, optimizing surgical visibility and instrument access while minimizing deformation of the sinonasal anatomy is paramount [30]. This approach is vital when selecting surgical strategies to mitigate postoperative olfactory impairment, while maintaining the objectives of the surgical intervention. The preservation of olfactory function may be achieved through conservative septal resection techniques that prioritize maintaining septal structural integrity.

## Supporting information

S1 Data (XLSX)
